# Cytoreductive Surgery plus Hyperthermic Intraperitoneal Chemotherapy for Management of Peritoneal Sarcomatosis: A Preliminary Single-Center Experience from Saudi Arabia

**DOI:** 10.1155/2016/6567473

**Published:** 2016-04-24

**Authors:** Ahmed Abu-Zaid, Ayman Azzam, Mohammed Abuzaid, Tusneem Elhassan, Naryman Albadawi, Lynn Alkhatib, Osama AlOmar, Abdullah Alsuhaibani, Tarek Amin, Ismail A. Al-Badawi

**Affiliations:** ^1^College of Medicine, Alfaisal University, Riyadh 11533, Saudi Arabia; ^2^Faculty of Medicine, Alexandria University, Alexandria 21526, Egypt; ^3^King Faisal Oncology Centre, King Faisal Specialist Hospital & Research Centre, Riyadh 11211, Saudi Arabia; ^4^Department of Obstetrics and Gynecology, King Fahad Medical City, Riyadh 11525, Saudi Arabia; ^5^Department of Obstetrics and Gynecology, King Faisal Specialist Hospital and Research Centre, P.O. Box 3354, Riyadh 11211, Saudi Arabia

## Abstract

*Aim.* To report our preliminary single-center experience with cytoreductive surgery (CRS) plus hyperthermic intraperitoneal chemotherapy (HIPEC) for management of peritoneal sarcomatosis (PS).* Methods.* Eleven patients were retrospectively analyzed for perioperative details.* Results.* Cytoreduction completeness (CC-0/1) was achieved in all patients with median peritoneal cancer index (PCI) of 14 ± 8.9 (range: 3–29). Combination cisplatin + doxorubicin HIPEC chemotherapy was used in 6 patients. Five patients received intraoperative radiation therapy (IORT). The median operative time, estimated blood loss, and hospital stay were 8 ± 1.4 hours (range: 6–10), 1000 ± 250 mL (range: 700–3850), and 11 ± 2.4 days (range: 7–15), respectively. Major postoperative Clavien-Dindo grade III/IV complications occurred in 1 patient and none developed HIPEC chemotherapy-related toxicities. The median overall survival (OS) and disease-free survival (DFS) after CRS + HIPEC were 28.3 ± 3.2 and 18.0 ± 4.0 months, respectively. The median follow-up time was 12 months (range: 6–33). Univariate analysis of several prognostic factors (age, gender, PS presentation/pathology, CC, PCI, HIPEC chemotherapy, and IORT) did not demonstrate statistically significant differences of OS and DFS.* Conclusion.* CRS + HIPEC appear to be feasible, safe, and offer survival oncological benefits. However, definitive conclusions cannot be deduced.

## 1. Introduction

Soft tissue sarcomas (STSs) are quite rare neoplasms accounting for roughly 1% of all adult solid malignancies [1]. Approximately 30% [2] to 36% [3] of all STSs originate in the retroperitoneum or abdominopelvic cavity. The natural biological behavior of STSs is characterized by an increased tendency for disease dissemination [4] and recurrence [5, 6]. Modes of disease dissemination include local invasion, peritoneal infiltration, blood-borne, and rarely lymph-borne spread [4]. Around 35% to 82% of all STSs will experience disease recurrence after the initial surgical management [5, 6]. The vast majority of these recurred STSs (80–90%) will progress and present as peritoneal sarcomatosis (PS)—multinodular intraperitoneal dissemination of STS [7]. This PS is especially true for abdominal/retroperitoneal STSs; however, trunk and limb STSs only exceptionally result in PS. Also, it should be recognized that PS may be the primary presentation in a proportion of patients [8].

Prognosis of patients with primary or secondary (recurrent) PS is generally poor with an estimated median overall survival ranging from 6 to 15 months [5, 9–11]. Current therapeutic modalities such as surgery, radiotherapy, and chemotherapy are largely ineffective against PS [8]. Therefore, an aggressive locoregional approach for management of patients with PS and no extraperitoneal disease has been suggested [8, 12]. There is a universally agreed upon consensus that aggressive locoregional management of PS requires a well-studied comparison between cytoreductive surgery (CRS) alone and combined modalities of CRS plus hyperthermic intraperitoneal chemotherapy (HIPEC) [13].

The combination of CRS plus HIPEC has been employed successfully in locoregional management of peritoneal mesothelioma [14] as well as peritoneal carcinomatosis arising from epithelial ovarian [15], nonepithelial ovarian [16], endometrial [17], appendiceal (pseudomyxomaperitonei) [18], colorectal [19], and gastric [20] cancer origins. The use of CRS plus HIPEC for management of primary or recurrent PS remains a subject of controversy [6, 8, 21–26].

The aim of this study is to report our single-center experience (feasibility, morbidity/mortality, and oncological outcomes) regarding the use of CRS plus HIPEC for management of patients with primary and recurrent PS.

## 2. Materials and Methods

The study took place at King Faisal Specialist Hospital & Research Centre (KFSH&RC), Riyadh, Saudi Arabia—a tertiary healthcare center. The study protocol was approved by the Research Advisory Council (RAC) and Institutional Review Board (IRB) at KFSH&RC, Riyadh, Saudi Arabia (RAC Project #22161039).

From November 2008 to October 2014, all patients with primary (first disease presentation) and secondary (recurrent disease presentation) PS managed by CRS plus HIPEC were retrospectively analyzed for perioperative (preoperative, operative, and postoperative) details.

Preoperative details included age, gender, presenting symptoms, PS presentation, Eastern Cooperative Oncology Group (ECOG) performance status, PS primary site of origin, PS pathology, and previous treatment (surgery, radiotherapy, and chemotherapy). Operative details included visceral surgical resections, cytoreduction completeness (CC), peritoneal cancer index (PCI), HIPEC chemotherapeutics, use of intraoperative radiation therapy (IORT), use of prophylactic (not routine) ureteral stents, operative time (OT), estimated blood loss (EBL), and hospital stay. Postoperative details included follow-up duration, 60-day morbidity (Clavien-Dindo surgical complications), 60-day mortality, 60-day readmission, adjuvant therapy (radiotherapy and/or chemotherapy), disease progression, site of recurrence (local, locoregional, distant, or combination), and current status (alive with disease, alive without disease, or dead).

In our tertiary healthcare center, intraoperative administration of HIPEC is optional. Patients were informed in detail about the current literature, benefits and risks of undergoing the standard treatment (CRS ± adjuvant therapy), or the optional recommended treatment (CRS + HIPEC ± IORT ± adjuvant therapy). Afterwards, patients were requested to sign a written consent regarding the desired treatment option.

Inclusion criteria for considering CRS plus HIPEC included (1) age below 75 years, (2) ECOG performance status ≤2, (3) satisfactory hematological, hepatic, coagulation, renal, and electrolyte profiles, (4) proven diagnosis of primary or secondary PS confirmed by imaging modality and/or intraoperative biopsy, (5) no evidence of PS distant extra-abdominopelvic metastatic foci to brain, lungs, liver, or bones, and (6) signed written informed consent by patients.

All operations were carried out by the same surgeons from Department of Surgical Oncology and Department of Obstetrics and Gynecology. IORT was performed by the same team from Department of Radiation Oncology.

Under general anesthesia, justified prophylactic (not routine) ureteral stents were inserted by the urology team at the beginning of the operation before carrying out CRS plus HIPEC procedure. Afterwards, a midline incision extending from xiphoid process to pubic tubercle was performed to completely explore the abdominopelvic cavity for PS. The extent of PS was evaluated intraoperatively using PCI [27]. CRS was performed as previously documented by Sugarbaker [12] and included multiple visceral resections directed towards optimal eradication of neoplastic foci from abdominopelvic cavity. After completion of CRS, assessment of residual tumors was determined intraoperatively using the standard CC scores, as documented by Sugarbaker [27]. CC-0 (no gross residual disease) score was regarded as complete cytoreduction, whereas CC-1 (up to 2.5 mm gross residual disease) score was regarded as near-complete cytoreduction. Only patients with CC-0 or CC-1 scores were considered for intraoperative HIPEC intervention.

Open-abdomen HIPEC technique was performed at the end of CRS. Abdominopelvic cavity was lavaged 15 times with 1 liter of normal saline prior to HIPEC. Two inflow drains were positioned below hemidiaphragms whereas two outflow drains were positioned in pouch of Douglas. All drains were connected to an extracorporeal closed sterile circuit in which a 2-liter perfusate was circulated by means of two peristaltic rollup pumps (one inflow and one outflow) at a flow rate of 2 L/min. HIPEC drugs were supplemented to the perfusate and allowed to circulate in abdominopelvic cavity for 90 min at 41.0–42.2°C. The heated perfusate plus chemotherapy (41.0–42.2°C) was achieved by a mean of heat exchanger connected to the sterile circuit. Intraperitoneal temperature was continuously checked by thermometers situated in abdominopelvic cavity to ensure maintenance of 41.0–42.2°C. Options of HIPEC drugs included combination of cisplatin (50 mg/m^2^) plus doxorubicin (15 mg/m^2^), single-agent melphalan (20 mg/m^2^), or single-agent mitomycin-c (15 mg/m^2^) as per the treating surgical oncology and medical oncology multidisciplinary team.

During HIPEC procedure, hemodynamic and cardiopulmonary parameters were continuously and carefully monitored. At the end of HIPEC procedure, abdominopelvic cavity was again lavaged 10–15 times with 1 liter of normal saline. A number of selected patients received IORT following HIPEC as deemed necessary by the treating surgical oncology and medical oncology multidisciplinary team.

At the end of CRS plus HIPEC plus/minus IORT procedure, ureteral stents were removed by the urology team and all patients were extubated, transferred to intensive care unit (ICU) for 1–3 days (median: 1 day), and afterwards transferred to the surgical ward for recovery.

Postoperative morbidity and mortality following CRS plus HIPEC were evaluated according to the Clavien-Dindo classification system for postoperative complications [28].

Following CRS plus HIPEC, some patients received postoperative adjuvant therapy (radiotherapy, chemotherapy, or both) as deemed necessary by the treating surgical oncology and medical oncology multidisciplinary team.

All patients were followed up regularly. No patient was lost during follow-up visits. During the first year following HIPEC, patients were followed up every 3 months. During the second year and afterwards, patients were followed up every 6 months. The follow-up work-up included routine physical examination, hematological profiles (complete blood count), biochemical profiles (electrolyte, renal, bone, hepatic, and coagulation), serum tumor markers, chest X-ray, whole-body computed tomography (CT) scan, or position emission tomography/CT scan (whenever deemed necessary).

The study endpoints were disease-free survival (DFS) and overall survival (OS). DFS was calculated from the day of CRS plus HIPEC to the time of local/distant disease progression or last date of follow-up, whichever comes first. OS was calculated from the day of CRS plus HIPEC to the time of death or last follow-up, whichever comes first. DFS and OS rates were calculated according to the Kaplan-Meier method and compared by using the two-tailed log-rank test. Univariate analysis was performed using the Cox proportional hazards model to predict prognostic variables (age, gender, PS presentation, PS pathology, CC, PCI, IORT, and HIPEC chemotherapeutics) of DFS and OS. All statistical analyses were performed using SPSS software version 19 for Windows (SPSS Inc., Chicago, IL, USA). For all purposes, *p* values <0.05 were regarded as statistically significant.

## 3. Results

Eleven patients met the study inclusion criteria. Patients' preoperative details are summarized in Table 1. There were 9 males and 2 females. Four and seven patients had PS at primary (first disease) and secondary (recurrent disease) presentations, respectively. Ten and two patients had abdominopelvis and extra-abdominopelvis PS sites of origin, respectively. PS histologies included 7 retroperitoneal liposarcomas and 4 retroperitoneal nonliposarcomas.

Patients' operative details are summarized in Table 2. CC-0 and CC-1 were achieved in 7 and 4 patients, respectively. The median PCI was 14 ± 8.9 (range: 3–29). IORT was performed in 5 patients. Reasons for using IORT during the same procedure included one or more of the following: anatomically locally advanced deep-seated invasion of retroperitoneum, psoas major muscle, renal capsule, trigone of urinary bladder and seminal vesicles, hemidiaphragm, external iliac, abdominal aorta, and inferior vena cava vicinities by the PS tumor residues, and hence making surgical dissection/resection of such tumor residues—to a greater degree—technically unfeasible with potential critical intraoperative morbidity/mortality.

Regarding patients' postoperative complications, 1 patient developed grade I lung atelectasis that was managed conservatively with chest physiotherapy. Three patients developed grade II postoperative complications (pneumonia, wound infection, and upper limb thrombosis) that were managed with conservative pharmacological treatments (antibiotics, antibiotics and wound dressing, and anticoagulation, resp.). Lastly, 1 patient developed grade IVa unilateral obstructive uropathy leading to acute renal failure and was managed with intensive care unit (ICU) admission, intravenous fluids, and temporary nephrostomy tube insertion.

Overall, there was no renal or hematological systemic toxicity.

Regarding patients' postoperative details, the median hospital stay was 11 ± 2.4 days (range: 7–15). The median follow-up time was 12 months (range: 6–33). Two and four patients received adjuvant radiotherapy and chemotherapy, respectively. The rates of 60-day readmission and 60-day mortality were zero. Six patients developed disease progression. Two patients died at 6 and 10 months after CRS plus HIPEC due to combined local and distant recurrences. Four patients were alive with disease at 12, 19, 27, and 30 months. Five patients were alive and disease-free without proof of recurrence at 34, 21, 13, 7, and 7 months.

**Figure 1:**
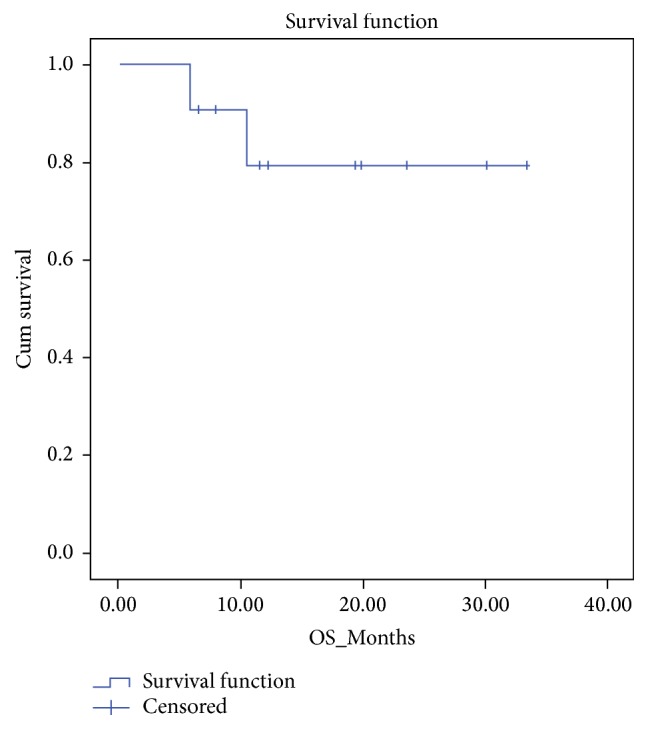
Kaplan-Meier figure for overall survival (OS) of all patients.

**Figure 2:**
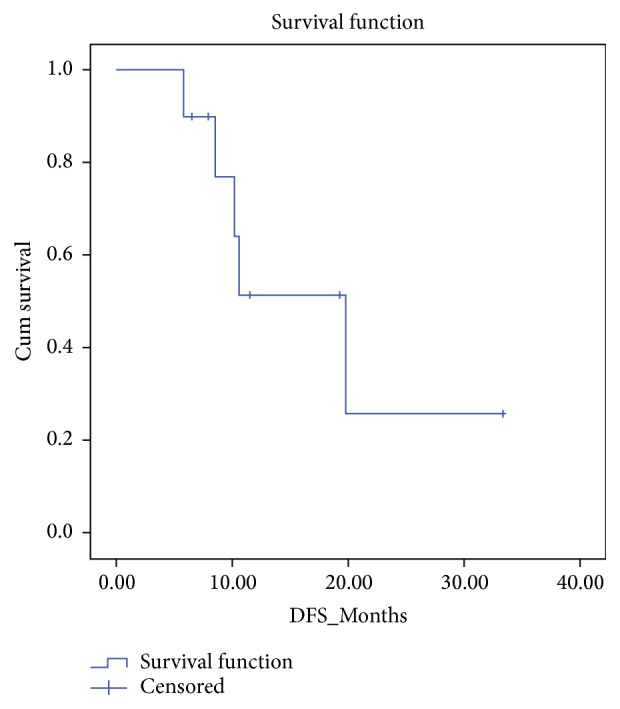
Kaplan-Meier curve for disease-free survival (DFS) of all patients.

Kaplan-Meier survival curves for OS and DFS are portrayed in Figures 1 and 2, respectively. For all patients, the median OS and DFS were 28.3 ± 3.2 (95% CI: 21.9–34.6) and 18.0 ± 4.0 (95% CI: 10.2–25.8) months, respectively.

Univariate analysis of the examined prognostic factors did not demonstrate statistically significant differences of OS and DFS.

## 4. Discussion

Although controversial, combination of CRS plus HIPEC has been advocated as an aggressive locoregional treatment for PS with fairly promising results [13]. This novel combined approach has been adopted by many oncological centers [6, 8, 21–26].  Table 3 summarizes a selected literature review on the role of CRS plus HIPEC for management of PS till the end of 2014.

Herein, from a developing country in Saudi Arabia, we presented our tertiary-care single-center experience of CRS plus HIPEC for management of primary and recurrent PS. Complete and near-complete cytoreduction with residual tumor nodules ≤2.5 mm was achieved in all 11 patients (100%). This percentage was fairly similar to the 68–100% reported in other studies [6, 8, 21–26]. Moreover, HIPEC was associated with tolerable grade III/IV morbidity (9.1%). This percentage was less than the 16–56% postoperative morbidity reported elsewhere [6, 8, 21–26]. Also, HIPEC was associated with neither intraoperative nor 60-day postoperative mortality. A recent systematic review of CRS plus HIPEC for management of PS showed that the perioperative mortality rate that ranged from as low as 0% to as high as 11% [6, 8, 21–26].

Complete/optimal cytoreduction (no microscopic residual disease)—irrespective of the number of multiple recurrences—remains the standard of care in management of PS and has been shown to be technically feasible and greatly influence the OS and DFS rates [6–10, 22, 29, 30]. Salti et al. [8] reported higher statistically significant mean DFS and OS in patients with optimal (CC-0) versus suboptimal (≥CC-1) cytoreduction (DFS: 27.3 versus 4.3 months, resp.; OS: 35.3 versus 5.3 months, resp.). In our study, the median OS and DFS rates did not differ between both groups and yet did not yield statistically significant differences on OS (*p* = 0.6) and DFS (*p* = 0.9).

The influence of PCI (disease volume) has been previously explored. Berthet et al. [29] reported higher 5-year OS rate in patients with PCI <13 than patients with PCI >13 (75% versus 12.8%, resp.). Conversely, our study (concerning DFS) as well as other several studies [6, 22, 24] failed to demonstrate any correlation between PCI and OS/DFS. This could be rationalized by the fact that the reported mean and/or median of PCI scores were most often low (<15), possibly implying the wise selection of patients with lower disease volumes.

The logic for employing HIPEC is centrally based on the direct hyperthermia-enhanced penetrative, synergistic, and cytotoxic effects of anticancer therapy on the neoplastic PS cells [4, 31]. In addition, as opposed to systemic chemotherapy, administration of intraperitoneal chemotherapy [8, 31] (1) effectively delivers higher locoregional drug concentrations without related undesirable systemic toxicities and (2) optimally sterilizes the surgical field against microscopic cancerous residues before development of postoperative adhesions and subsequent entrapment of cancerous cells in scar tissues—the most substantial factor prompting PS recurrence [6–10, 22, 29, 30]. The aforementioned advantages of HIPEC enable the combination of CRS plus HIPEC to mount as potentially noteworthy treatment for PS.

The choice of intraperitoneal chemotherapy drugs should be justified by confirmed scientific evidence of hyperthermia-improved effects of the chosen intraperitoneal chemotherapies. Examples of such chemotherapies include cisplatin [32], mitomycin-c [33], doxorubicin [34], mitoxantrone [35], and melphalan [36, 37]. In our study, mitomycin-c was used only in 1 patient which was the first early case of HIPEC. Reasons for its use included (1) proven profile of hyperthermia-enhanced cytotoxic effects [33], (2) good pharmacokinetic profile for locoregional administration, despite poor effectiveness in sarcoma, and (3) limited availability of HIPEC drugs at the time of procedure at our institute.

In our study, age, gender, PS presentation, and PS pathology did not influence OS or DFS on univariate analysis. Similar results were obtained elsewhere [6, 8, 22, 23].

Adjuvant therapies such as systemic chemotherapy and external beam radiation therapy (EBRT) do not appear to provide any clinical benefits [6], as STSs are largely chemoresistant and radio-resistant tumors [38, 39]. Moreover, additional novel therapeutic modalities such as preoperative EBRT combined with postoperative brachytherapy [40], IORT [41, 42], and photodynamic therapy [43, 44] have been suggested to enhance the life expectancy of these patients, but there are no solid clinical results to offer solid conclusions.

Previous studies showed that application of IORT (dose: 8.75–30 Gy) is associated with improved 5-year OS (45–64.8%), 5-year local disease control (40–62%), and 5-year DFS (28–55%) in patients with primary advanced or recurrent STSs [41, 42, 45, 46]. To the best of our knowledge, none of the previous CRS plus HIPEC studies used IORT in the same procedure; hence, this is the first ever study of such combination (CRS plus HIPEC plus IORT) in the literature. At present, there is limited effectiveness of the available therapies for PS [22]. Moreover, there are no universally agreed upon guidelines for management of PS [6], and consequently there is lack of adequate studies to conclude solid recommendations. That being said, the process of identifying novel treatment modalities for PS is continuingly evolving. In our study, the novel combination modality (CRS plus HIPEC plus IORT) was carried out in an optimistic attempt to provide synergistic anticancer treatment, achieve better aggressive locoregional disease control especially in the setting of neighboring technically unresectable tumor residues, and ultimately yield improved survival benefits in such patients. However, IORT did not influence OS or DFS on univariate analysis. More studies regarding this novel combination are needed and this is an interesting area for future research.

Limitations to this study include the following: retrospective study design, relatively small sample size, relatively short period of follow-up, and lack of consistent therapy and control group; all of which limitations were previously documented in earlier studies [6, 8, 21–26].

## 5. Conclusion

CRS plus HIPEC appear to be feasible and safe and offer survival oncological benefits. Despite the current results being encouraging, definitive conclusions regarding the role of HIPEC in providing locoregional disease control cannot be deduced. The novel firstly introduced addition of IORT to CRS plus HIPEC during the same procedure did not significantly influence OS or DFS on univariate analysis. The impact of HIPEC following CRS remains questionable and still has to be further investigated in randomized controlled large-sized multi-institutional studies. Until then, cytoreduction to no macroscopic disease remains the standard of care in management of PS, and the administration of HIPEC remains a topic of debate with no conclusive recommendations.

## Figures and Tables

**Table 1:** Preoperative details of patients.

Variable	*n* (%)
Median age ± SD (range)	46 ± 10.9 (19–57)
Median ECOG performance status score ± SD (range)	1 ± 0.6 (0–2)
Gender	
Male	9 (81.8%)
Female	2 (18.2%)
PS presentation	
Primary (first disease)	4 (36.4)
Secondary (recurrent disease)	7 (63.6)
Site of origin	
Abdominal/pelvic	9 (81.8)
Extra-abdominal/pelvic	2 (18.2)
*Both cases were liposarcomas from lower limb, specifically thigh and calf muscles*	
Histology (pathology)	
Retroperitoneal liposarcomas	7 (63.6)
Retroperitoneal non-liposarcomas	4 (36.4)
Leiomyosarcoma	1 (9.1)
Ewing's Sarcoma	1 (9.1)
GIST (fundus and body origins)	2 (18.2)
Previous treatment	
Surgery	7 (63.6)
Radiotherapy	2 (18.2)
Chemotherapy	4 (36.4)
Symptoms	
Asymptomatic	1 (9.1)
Flank pain	2 (18.2)
Abdominal pain	8 (72.7)
Increased abdominal circumference	2 (18.2)
Early satiety, nausea, and vomiting	2 (18.2)
Weight loss	3 (27.3)

SD: standard deviation; PS: peritoneal sarcomatosis; ECOG: Eastern Cooperative Oncology Group; GIST: gastrointestinal stromal tumor.

**Table 2:** Operative details of CRS plus HIPEC.

	*n* (%)
Viscera resected	
Appendectomy	4 (36.4)
Cholecystectomy	7 (63.6)
Splenectomy	2 (18.2)
Distal pancreatectomy	1 (9.1)
Omentectomy	4 (36.4)
Peritonectomy	5 (45.5)
Anterior parietal peritonectomy	3 (27.3)
Pelvic peritonectomy	2 (18.2)
Urinary bladder dissection	1 (9.1)
Diaphragm resection	1 (9.1)
Small bowel resection	4 (36.4)
Large bowel resection	8 (72.7)
Low anterior resection	1 (9.1)
TAH + BSO	2 (18.2)
Median enteric anastomosis (range)	1 (1–3)
CC	
CC-0	7 (63.6)
CC-1	4 (36.4)
Median PCI ± SD (range)	14 ± 8.9 (3–29)
HIPEC chemotherapeutic	
Cisplatin plus doxorubicin	6 (54.5)
Melphalan	4 (36.4)
Mitomycin-c	1 (9.1)
Intraoperative radiation therapy (IORT)	5 (45.5)
Median operative time ± SD (range)	8 ± 1.4 hr (6–10)
Median EBL ± SD (range)	1000 mL ± 250 (700–3850)
Intraoperative morbidity	0
Intraoperative mortality	0

SD: standard deviation; CC: cytoreduction completeness; PCI: peritoneal cancer index; HIPEC: hyperthermic intraperitoneal chemotherapy; TAH + BSO: total abdominal hysterectomy and bilateral salpingo-oophorectomy; EBL: estimated blood loss.

**Table 3:** Selected literature review on CRS + HIPEC for management of PS (till end of 2014).

Ref	Author	Year	*n*	HIPEC Chemo	CC-0/1	PCI	Median FU	Median DFS	Median OS	Median 5-year OS	Mortality	Morbidity	HS	Pathology
[6]	Rossi et al.	2004	60	Cis + dox	68	7.7	28	22	34	38	0	33	12	GIST = 14, RPS = 34, and LMS = 12

[21]	Lim et al.	2007	19	Cis	95	NR	NR	4.4	16.9	NR	0	16	15	GIST = 15, DSRCT = 3, and LS = 1

[21]	Lim et al.	2007	9	Cis + mitox	100	NR	NR	2.3	5.5	NR	11	44	16	GIST = 2, DSRCT = 2, LS = 1, sarcomatoid = 1, and unclassified = 3

[22]	Baratti et al.	2010	37	Cis + dox or MMC	84	14.7	104	12.1	26.2	24.3	2.7	21.6	NR	RPS = 13, uterine = 11, GIST = 8, DSRCT = 3, myxofibrosarcoma = 1, and LMS = 1

[8]	Salti et al.	2012	13	Cis + dox	70	12.1	12	11	12	NR	0	15.4	NR	LS = 8, pleomorphic sarcomas = 2, angiosarcoma = 1, and carcinosarcoma = 2

[23]	Baumgartner et al.	2013	17	Cis, dox or MMC	100	6^#^	17.4	17.2	22.6	35	0	23.5	8	SCS = 4, LS = 5, LMS = 3, GIST = 2, and others = 3

[24]	Sommariva et al.	2013	15	Cis + dox or cis + MMC	100	5.5	28	15	27	29	0	46.6	NR	LS = 4, GIST = 2, LMS = 4, histiocytoma fibrous malignant = 1, DSRCT = 1, SCS = 1, schwannoma = 1, and stromal sarcoma = 1

[25]	Randle et al.	2013	10	Cis or mitox	60	NR	84.8	NR	21.6	50	0	NR	10^$^	DSRCT = 1, fibrosarcoma = 1, LMS = 2, SS = 4, and hemangiopericytoma = 2

[26]	Bryan et al.	2014	18	MMC ± mitox	72.2	NR	NR	NR	40	56^*∗*^	5.6	33.3	8	GIST before era of TKI

	Current study	2015	11	Cis + dox or MMC or mel	100	14	12	18	28.3	NR	0	18.2	11	LS = 7, GIST = 2, uterine LMS = 2, and ES = 1

Ref: reference; HIPEC: hyperthermic intraperitoneal chemotherapy; Chemo: chemotherapy; CC: cytoreduction completeness; PCI: peritoneal cancer index; FU: follow-up; DFS: disease-free survival; OS: overall survival; HS: hospital stay; cis: cisplatin; dox: doxorubicin; mitox: mitoxantrone; MMC: mitomycin-c; Mel: melphalan; GIST: gastrointestinal stromal tumor; RPS: retroperitoneal sarcoma; LMS: leiomyosarcoma, LS: liposarcoma; DSRCT: desmoplastic small round cell tumor; SS: spindle-cell sarcoma; SCS: synovial cell sarcoma; TKI: tyrosine kinase inhibitor; NR: not reported.

^*∗*^3-year survival.

^$^mean.

^#^SPCI (simplified peritoneal cancer index).
